# Manufacturing and Characterization of 18Ni Marage 300 Lattice Components by Selective Laser Melting

**DOI:** 10.3390/ma6083451

**Published:** 2013-08-13

**Authors:** Nicola Contuzzi, Sabina L. Campanelli, Caterina Casavola, Luciano Lamberti

**Affiliations:** Dipartimento di Meccanica, Matematica e Management, Politecnico di Bari, Viale Japigia 182, Bari 70126, Italy; E-Mails: campanel@poliba.it (S.L.C.); casavola@poliba.it (C.C.); lamberti@poliba.it (L.L.)

**Keywords:** SLM, steel powder, lattice structures, mechanical tests, finite element analysis

## Abstract

The spreading use of cellular structures brings the need to speed up manufacturing processes without deteriorating mechanical properties. By using Selective Laser Melting (SLM) to produce cellular structures, the designer has total freedom in defining part geometry and manufacturing is simplified. The paper investigates the suitability of Selective Laser Melting for manufacturing steel cellular lattice structures with characteristic dimensions in the micrometer range. Alternative lattice topologies including reinforcing bars in the vertical direction also are considered. The selected lattice structure topology is shown to be superior over other lattice structure designs considered in literature. Compression tests are carried out in order to evaluate mechanical strength of lattice strut specimens made via SLM. Compressive behavior of samples also is simulated by finite element analysis and numerical results are compared with experimental data in order to assess the constitutive behavior of the lattice structure designs considered in this study. Experimental data show that it is possible to build samples of relative density in the 0.2456–0.4367 range. Compressive strength changes almost linearly with respect to relative density, which in turns depends linearly on the number of vertical reinforces. Specific strength increases with cell and strut edge size. Numerical simulations confirm the plastic nature of the instability phenomena that leads the cellular structures to collapse under compression loading.

## 1. Introduction

Layer manufacturing (LM) technologies produce 3D physical parts directly from CAD solid models. Since 3D Systems Inc. introduced the first rapid prototyping (RP) system (stereolithography, SL) in the late 1980s, many layer manufacturing technologies and systems have been developed. These technologies, initially directed towards the production of prototypes, have recently been used to fabricate tooling and functional parts [1,2].

Selective Laser Melting (SLM) is probably the most rapidly growing technique in RP and LM technologies. This is due in most part to the possibility of creating metal parts with complex shape and intrinsic engineered characteristics. Furthermore, SLM can produce parts whose mechanical properties are comparable with those of components made with traditional processes. Most SLM literature focused on the optimization of the technological process to obtain almost full density of parts and good mechanical properties of the bulk materials (see, for example, [2,3]).

More recently, the use of SLM has been extended to the fabrication of low-density lattice structures [4,5,6,7,8,9,10,11,12,13,14,15,16,17,18,19,20]. Reinhart *et al.* [5] showed that additive layer manufacturing entails many advantages in the production of lightweight components. This ability derives from the large freedom in terms of geometry that can be achieved with respect to conventional manufacturing processes. Consequently, the efficient utilization of material allows good lightweight components to be designed.

Smith *et al.* [6] investigated the quasi-static and blast response of 316L stainless steel lattice structures. In particular, they compared the compression behavior of a body-centered cubic (BCC) structure and a similar structure including vertical pillars (BCCZ) built by SLM. It appears that adding a vertical strut into the BCC structure yields drastic improvements in mechanical properties of the structure.

Shen *et al.* [7] manufactured BCC octahedral and BCCZ pillar-octahedral SLM micro-lattice structures to be used in the design of sandwich strut core materials. Tensile tests performed on these parts showed that mechanical properties do not vary significantly over the range of fabricated angles considered in the study. Compression tests done on blocks showed that micro-lattice structures can absorb high energy due to the formation of plastic hinges at the junction points within the structure.

Cellular metal structures (CMS) have become widely diffused in lightweight structure constructions because of their excellent characteristics such as low density, high specific strength against compression, high bending stiffness, good absorption of energy, good thermal and acoustic properties [8]. The most relevant applications of CMS are in the fields of thermotechnics (e.g., heat exchangers), reconstructive surgery, chemical industry, automotive constructions and aerospace industry.

The increasing demand of CMS for engineering applications has led to develop a relevant number of fabrication processes. From this stand point, cellular materials can be classified in terms of variability in cell size (*i.e.*, regular or stochastic), topology of pores (*i.e.*, open or closed), relative density of structure and cell size. However, manufacturing processes currently available for cellular materials do not allow part mesostructure, material composition and part macrostructure to be fully controlled [9].

Stochastic cellular structures such as foams are very suitable for very particular surface applications, acoustic insulation; furthermore, they can absorb considerable amount of energy [21]. Metallic foams are currently studied with a great deal of interest because of their very low density. These materials can be produced with either closed- or open-cell structure. Fairly regular cell arrangements are obtained in structures including hollow spheres, which are often called syntactic foams. However, the main limitation of stochastic cellular structures is the lack or even the total absence of freedom left to the designer in selecting cell structure topology.

Periodic structures such as honeycombs or lattice strut structures have better mechanical properties (energy absorption, strength and stiffness), carry higher loads and can better drive thermal flows than stochastic cellular structures. For example, structural performance of lattice strut structures with less than 5% density was proven to be up to three times higher than that of stochastic foams [22]. Such a large improvement in strength depends on the fact that foam structural behavior is driven by cell wall bending while lattice strut elements may be subject only to tension or compression in the direction of the element axis. For this reason, lattice materials received more attention than foams. However, manufacturing processes still are fairly complicated thus limiting the applications of these foams in terms of freeform design and selection of materials.

Lattice strut-core structures, like pyramidal or tetrahedral structures, are usually made of highly-ductile alloys. A folding process can be utilized to bend elongated hexagonal (tetrahedral lattice strut) or rhomboidal (pyramidal lattice strut) sheets in order to create a single layer strut lattice. Folding can be realized node row by node row by means of a paired punch and die tool with sheets folded so to form regular tetrahedrons. Cores may be bonded to facesheets by conventional methods such as brazing or laser welding.

The continuously growing number of applications of cellular structures pushed towards increasing the speed of manufacturing processes yet without sacrificing mechanical properties. Furthermore, designers should be influenced the least as it is feasible by manufacturing constraints. Selective Laser Melting, seen as a technological process to produce cellular structures, has the clear advantage to let designers completely free in defining part geometry thus making it possible to build very complicated 3D parts. Furthermore, SLM can allow the production cycle to be simplified as well as to improve thermal management of the manufacturing process.

In spite of the above mentioned advantages, technical literature does not present many systematic investigations on properties of SLM-built lattice structures to assess relative merits of different cell topologies in terms of relative density, thermal dissipation and mechanical strength. For this reason, the article presents a comprehensive study on the performance of micro-lattice structures fabricated via SLM using 18Ni Marage 300 powder. The choice of this material is dictated by its high suitability for fabrication of injection molding tools and inserts. In fact, SLM can directly create any kind of cellular structure inside the part currently manufactured without requiring any additional manufacturing operation and regardless of the complexity of the part. This ability turns extremely useful in the production of molding tools with complex external design and conformal cooling channels. By adopting cellular type interiors, it is possible to significantly reduce the production cycles and contribute to the thermal management of the molding process [9]. Basic and enhanced micro-structure topologies are considered. In particular, experimental tests and finite element simulations are performed in order to compare the compression behavior of micro-lattice structures and micro-lattice structures reinforced by bars.

## 2. Choice and Design of Lattice Structures

In order to select the best unit-cell topology, the most common design concepts (pillar texile, diamond lattice, diamond texile, kagome, pillar octahedral, square collinear) were compared in terms of relative density *ρ*, area density factor *β*, Von Mises maximum stress σ_VM_ developed in the cell structure and compression failure load R. The relative density *ρ* is defined as the ratio between the volume V_cell_ actually filled by the cell structure and the total volume V_C_ enclosed by the cell. That is:

(1) $$\rho =\frac{{\text{V}}_{\mathrm{cell}}}{{\text{V}}_{\text{c}}}$$

The area density factor *β* is defined as the ratio of the surface S_cell_ of the cell filled volume to the cell volume V_cell_. That is:

(2) $$\beta =\frac{{\text{S}}_{\mathrm{cell}}}{{\text{V}}_{\mathrm{cell}}}$$

Compact heat exchangers (CHE) are characterized by values of *β* greater than 0.7 mm^2^/mm^3^. CHE have significantly higher efficiency, smaller volume and lower weight than traditional heat exchangers. Faster heat exchange is allowed by the much larger surface exposed to fluid. Furthermore, CHE can efficiently work at higher pressures and even very low difference in temperature between hot and cold fluids. Conversely, heat exchange efficiency of traditional heat exchangers rapidly drops down if temperature difference goes below 5 °C [23,24].

Nonlinear finite element analysis, including both material and geometric nonlinearity (for example, roughness was included as geometric nonlinearity in the FE model in fashion of surface waviness with an amplitude corresponding to R_a_), was utilized to determine the maximum Von Mises σ_VM_ stress developed in the cell and the compression failure load for each cell topology. More details on FE modeling will be given in Section 4.

**Figure 1:**
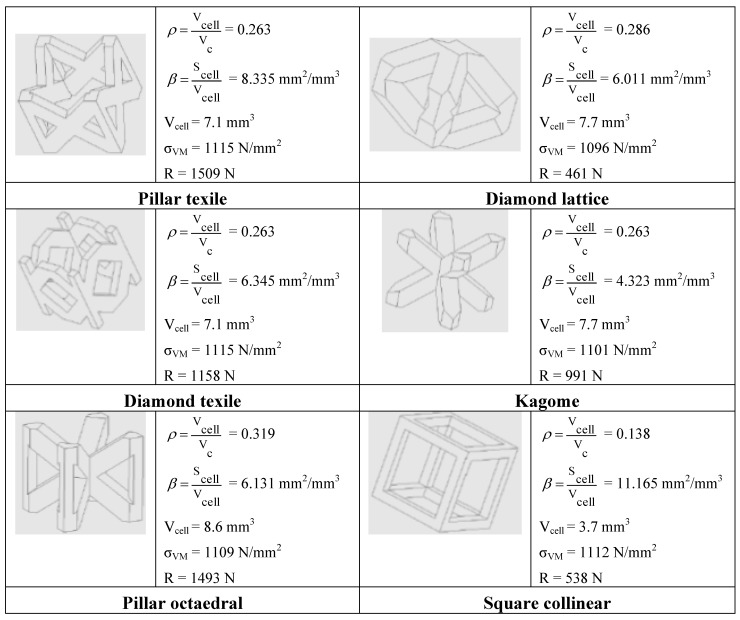
Comparison of performance of the most common cell topologies.

Figure 1 compares the most commonly used cell topology designs with respect to the four above mentioned performance indicators (*ρ*, *β*, σ_VM_ and R). In order to carry out a realistic comparison, the cell size (3 mm) and the thickness of single strut edge (700 μm) were always the same for all of the investigated topology concepts. Since struts with square cross section are selected, STL files include less triangles than in the case of circular cross sections and hence are smaller in size and more easily manageable in the processing phase.

It can be seen from the table that the maximum stress developed in the cell is rather insensitive to cell geometry: in fact, the average value of maximum stress is 1108 MPa (about 90% of the ultimate strength of the 18 Marage 300 material) with a standard deviation of only 8 MPa, that is less than 0.7% of average stress.

The square collinear concept has the lowest relative density overall (*i.e.*, 0.138) and the highest area density factor (11.165 mm^2^/mm^3^). However, it has very small compressive strength. This suggested that we should not consider the square collinear concept for structural purposes.

The best combination of properties is exhibited by the pillar texile concept, which is the second best structure in terms of relative density (0.263 *vs.* 0.138) and area density factor (8.335 mm^2^/mm^3^ *vs.* 11.165 mm^2^/mm^3^) and the best concept in terms of compressive strength (1509 N). The pillar octahedral concept is quite similar to pillar texile in terms of mechanical strength but definitely less efficient in terms of relative density and heat exchange properties.

**Figure 2:**
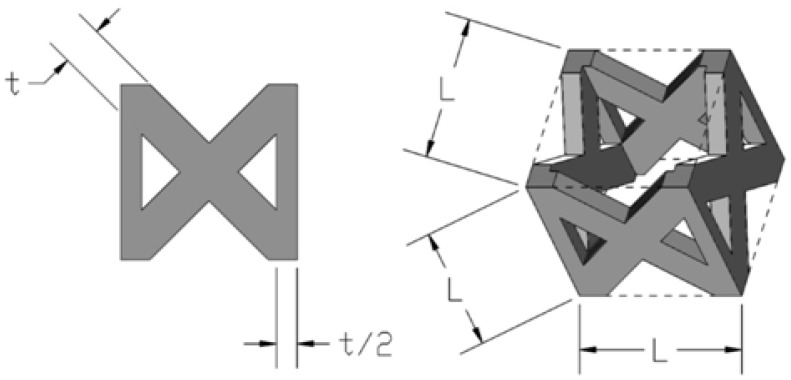
Schematic illustration of the pillar texile unit cell and main geometric dimensions.

The pillar texile lattice structure comprised of four vertical strut columns and four couples of struts inclined at ±45° with respect to cell axes of symmetry was hence selected. Figure 2 shows the unit cell of the lattice structure chosen in this study.

The volume of the struts contained in the unit cell can be calculated as follows:

(3) $${\text{V}}_{\text{L}}=(1+4\sqrt{2})\cdot \text{L}\cdot {\text{t}}^{2}-(5+2\sqrt{2})\cdot {\text{t}}^{3}$$

where L is the length of the base side of the unit cell (*i.e.*, the cell size) and t is thickness of the single strut edge. Since the unit cell volume is V_c_ = L^3^, the relative density can be computed as follows:

(4) $$\rho =\frac{{\text{V}}_{\text{L}}}{{\text{V}}_{\text{c}}}=\frac{(1+4\sqrt{2})\cdot \text{L}\cdot {\text{t}}^{2}-(5+2\sqrt{2})\cdot {\text{t}}^{3}}{{\text{L}}^{3}}$$

## 3. SLM Setup and Material

### 3.1. SLM Setup

Selective Laser Melting (SLM) is a solid freeform fabrication process where 3D parts are built layer by layer. Each layer of powder is molten by a high energy laser beam. The machine used in this research to perform experiments was equipped with a nanosecond Nd:YAG laser source (Rofin, Germany) pumped by laser diodes, and a scanning head with deflecting mirrors to direct the laser beam toward the scanner head lens. The laser can operate in both continuous and pulse mode: the maximum power is respectively 100 W and 20 W. The continuous mode is characterized by a laser spot diameter of 200 μm while the diameter of the laser spot in the pulse mode is 70 μm. In this work, the continuous mode was used. The laser beam was directed onto the powder surface by means of scanning mirrors in order to draw selectively every layer of the powder.

**Figure 3:**
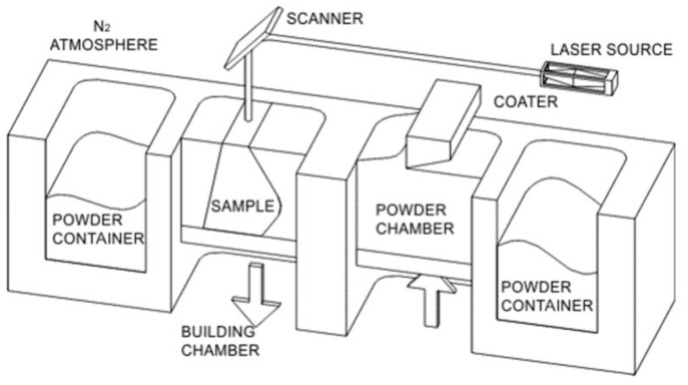
Schematic of the Selective Laser Melting equipment.

The powder deposition system (see the schematic shown in Figure 3) consisted of a powder platform, a working platform, and a coater, that deposited powder layers with thickness of 30 µm in only one direction. The powder chamber was filled with nitrogen to avoid oxidation of the parts and to reduce the initial oxygen level at 0.8%.

A previous study [25] showed that appropriate scanning patterns can yield an appreciable reduction in thermal deformations. Therefore, in order to produce parts with low residual stresses, the laser scanning strategy was optimized by dividing the part area in small square sectors (islands) of 5 mm × 5 mm. Furthermore, a random scanning sequence was chosen to melt each sector.

SLM process parameters were optimized in order to maximize the relative density of the manufactured parts. The scanning speed was set to 180 mm/s and the laser power to 100 W. This allowed to obtain a relative density (*ρ_r_*) of manufactured parts up to 99%, an ultimate tensile strength (*UTS*) of 1133 ± 33 MPa, an average surface roughness (R_a_) of 15 μm, and a hardness of 31 HRC. These values are consistent with those found in [3].

**Figure 4:**
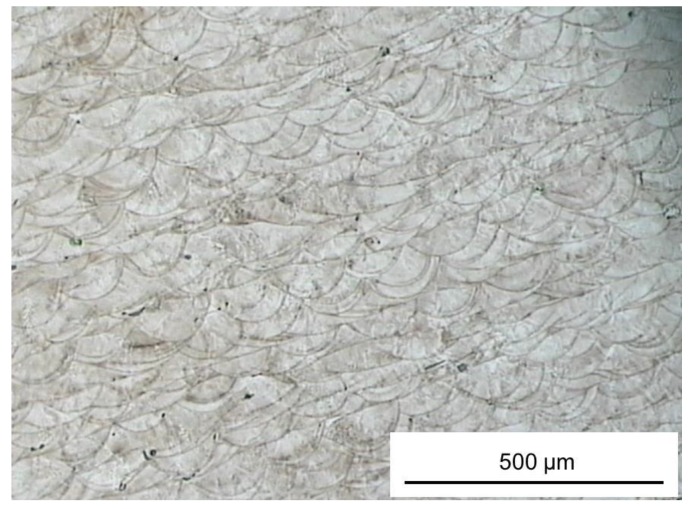
Micrographs of part cross-sectional area recorded at 100× magnification.

Information on the quality of the actual structure of the built layers can be gathered from Figure 4 that shows a micrograph of a cross-section captured by an optical microscope at 100× magnification. The metal powder appears to be completely fused and there are molten/resolidified zones approximately of parabolic shape. Because of the random scanning strategy and laser tracks overlapping, the resulting structure is characterized by very low porosity.

### 3.2. Materials

**Figure 5:**
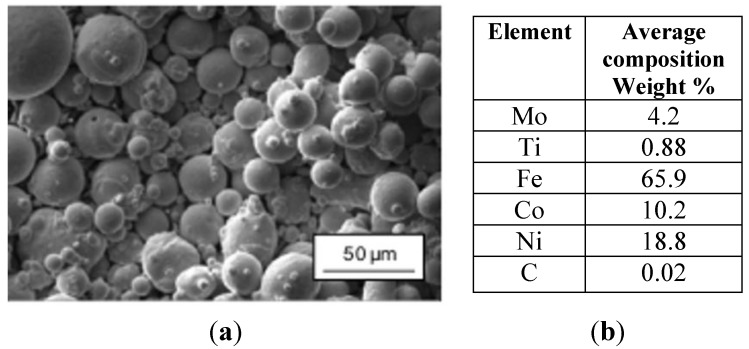
(**a**) SEM image of the CL50WC powder (1000× magnification); (**b**) Chemical composition of the powder.

In this work, the CL50WC powder (supplied by Concept Laser GmbH, Germany), with the typical composition of maraging steels, reinforced with cobalt particles, was used. More specifically, the composition of the powder is very close to the 18Ni Marage 300 steel. The powder particles were spherical with average size of 40 μm. Figure 5a shows Scanning Electron Microscope images of the powders. The chemical composition, studied by means of Energy Dispersive X-ray (EDX) analysis, is reported in Figure 5b.

The 18Ni Marage 300 steel has excellent mechanical properties, high yield stress and tensile strength, high toughness, ductility and impact strength, high fatigue limit, high compressive strength, hardness and wear resistance (this property makes it very suitable for tool machining), excellent characteristics after heat treatment, good machinability, high resistance to crack propagation, easy hot or cold molding [26,27].

## 4. Mechanical Characterization of the Micro-Lattice Structures

### 4.1. Design Concepts

Preliminary experiments were conducted in order to assess the capacity of SLM process to create lattice structures including strut elements as small as possible. The experiments served to find the minimum dimensions of the lattice structure that allow cell geometry to be realized without defects. Results showed that the minimum size of strut edge *t* must be 500 µm for the samples with cell size *L* = 2 mm, and 700 µm for the samples with *L* = 3 mm, respectively.

A more detailed experimental campaign was then carried out in order to compare two design concepts: (i) *Design A* with cell size *L* = 3 mm and size of strut edge *t* = 700 μm; (ii) *Design B* with cell size *L* = 2 mm and size of strut edge *t* = 500 μm. Overall dimensions (*D*) for designs A and B, respectively, are 24 × 24 × 15 mm^3^ and 16 × 16 × 16 mm^3^. Relative density is 0.2456 for design A while design B has a relative density of 0.3562.

**Figure 6:**
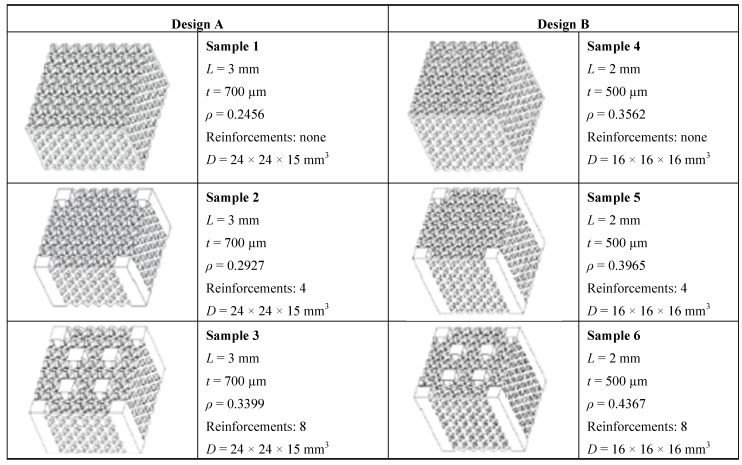
Schematic of optimized and alternative cell topologies including vertical reinforcements.

Besides the base designs A and B that reproduce the pillar texile topology, other samples, always with cell sizes of 2 or 3 mm, were studied. These samples, shown in Figure 6, included vertical reinforcements to improve the overall stiffness of the structure. The dimensions of the cross-sectional area of vertical struts are equal to those of the cell size. Samples 2 and 5 represent structures reinforced only at the four corners. Samples 3 and 6 represent structures reinforced at the four corners and at the centers of the diagonals. The values of relative density, cell size and edge size of strut also are indicated in the figure.

Figure 6 shows that the relative density of samples changes between 0.2456 and 0.4367. As expected, the introduction of reinforces leads to increase relative density.

### 4.2. Compression Tests

**Figure 7:**
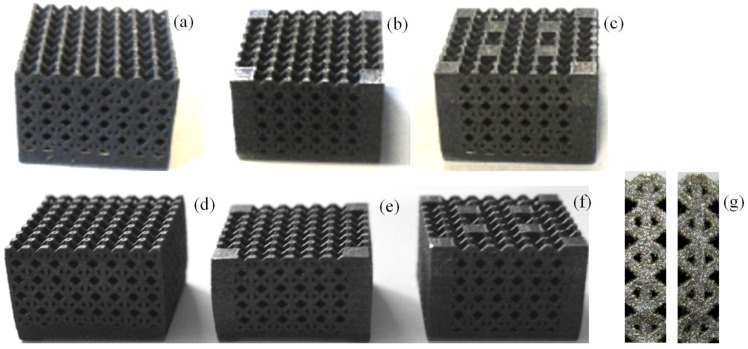
Samples 1, 2 and 3 built by SLM (Design A specimens): (**a**) Base structure; (**b**) Structure with 4 reinforcement columns; (**c**) Structure with 8 reinforcement columns; (**d**) Deformed base structure; (**e**) Deformed 4-column reinforced structure; (**f**) Deformed 8-column reinforced structure; (**g**) Typical collapse mode of struts observed in the experiments.

Figure 7a–c shows design A type samples 1, 2 and 3 manufactured by SLM. Figure 7d–f shows the corresponding deformed specimens. Similar images were obtained for design B type samples and hence are omitted for the sake of brevity. Typical collapse modes of struts are shown, for example, in Figure 7g: in particular, this detailed view shows elements located near reinforcement columns.

The six cell variants considered in this study were all tested under uniaxial compression. Mechanical tests were performed under displacement control with a Instron 4467 machine equipped with a 250 kN load cell; end-shortening was supplied to the specimen by setting compression speed to 0.5 mm/min. Five specimens were built and tested for each design variant to obtain statistically significant results. The test protocol was selected according to the collapse mode most likely to occur for the present structures that somehow resemble open-cell foams. Following the theory reported in the classical textbook by Gere and Timoshenko [28], if the lattice structure is made of a material with positive strain hardening rate, it starts to plastically deform and keeps to withstand increasing levels of stress due to the hardening of the struts that eventually collapse by plastic buckling. In view of this, in the present study, samples were loaded up to the limit of inelastic buckling and compression tests were terminated at 2.5 mm end-shortening. Strut collapse modes observed experimentally (see, for example, the detailed view in Figure 7g) are consistent with the theoretical collapse behavior described above.

**Figure 8:**
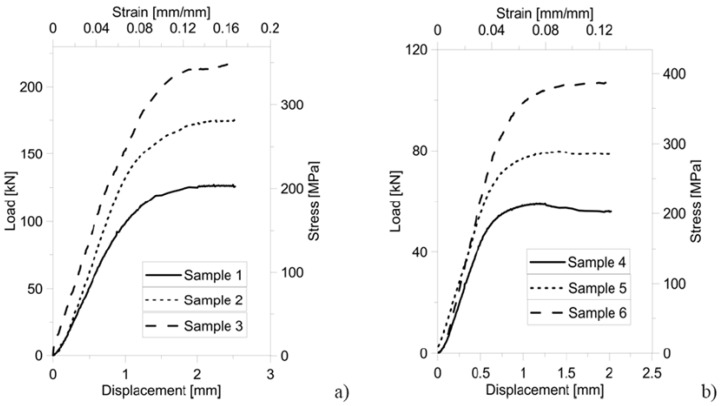
Load-displacement curves for design A type samples (**a**) and design B type samples (**b**).

Figure 8a,b shows the load-displacement/stress-strain curves, respectively, for design A and B type samples. Following literature, the stress was determined as the ratio between the applied load measured by the load cell and the nominal cross-section of the specimen; strain was determined as the ratio between the measured shortening and nominal height of the specimen. The inelastic buckling limit load corresponds to the peak force recorded in the load-displacement curve (see Figure 8). Values of stress and strain derived from the load-displacement curves recorded experimentally also are reported in the figure. Since the statistical dispersion of experimental data gathered for each design variant was within ±5%, the relative behavior of different samples can be reliably assessed from the curves shown in Figure 8.

Figure 8 demonstrates that by introducing the vertical reinforcements (*i.e.*, for design A type specimens, going from Sample 1 to Sample 3, and from Sample 4 to Sample 6 for design B type specimens) it is possible to increase the load carrying capability of the micro-lattice truss structure by a factor almost equal to 2. Remarkably, the same result was achieved independently of cell dimensions. Although the higher load carrying capability entailed heavier cell structures, it should be noted that by adding reinforcement struts into the cell structure the specific strength of the structure can increase more significantly. In fact, while relative density became only 38.3% or 22.6% higher than for the base (*i.e.*, unreinforced) structure, respectively, for design A and B samples, the load carrying capability of the cell structure even increased by 70%–80% (see Figure 8 and peak strength values listed in Table 1).

**Table 1:** Comparison of strength peaks measured experimentally and computed by FE analysis.

Sample	Peak strength [MPa]		
	Experiments	FE–MELAS	FE–MISO
1	206.0	195.7	211.1
2	285.6	266.7	280.3
3	351.8	327.3	341.4
4	97.0	104.4	117.4
5	130.6	136.0	147.4
6	175.4	163.7	174.4

Stress peak values change almost linearly with relative density: the correlation coefficient computed for the linear fitting is 0.9964 and 0.9917, respectively, for designs A and B specimens. Such a behavior was seen for all design variants. Maximum stress values recorded for design A type specimens are just 10% different from their counterpart for design B. Therefore, design A type specimens undergo the same level of stress as design B type specimens but their relative density is on average 30% lower than for design B.

In summary, design A allows the specific strength of the micro-cell structure to be significantly increased with respect to design B. This can be explained by considering that cell failure is driven by buckling. This phenomenon depends on the thickness and length of the strut elements of which the cell structure is comprised. Struts included in design A type samples are 1.4 times thicker than in the case of the samples falling in the design B class (*i.e.*, 700 μm *vs.* 500 μm) but also 1.5 times longer than for design B type specimens (*i.e.*, 3 mm *vs.* 2 mm). The increasing in buckling strength with thickness (strength changes approximately as the fourth power of thickness [29]) is more significant than the reduction of buckling strength introduced by the presence of longer strut elements (buckling load changes quadratically with length).

The plastic nature of the failure mechanisms observed in the experimental tests is confirmed by the fact that the plastic plateau present in the load-displacement curves is less pronounced for design A type specimens. Furthermore, for a given specimen type, the plateau becomes less significant as the number of reinforcement columns included in the cellular structure increases. Failure begins to develop locally in correspondence of the vertical beams that mostly carry the compressive load. As the peak load is reached, diffuse plasticization of the material occurs and finally structure crushes down.

### 4.3. Finite Element Analysis

**Figure 9:**
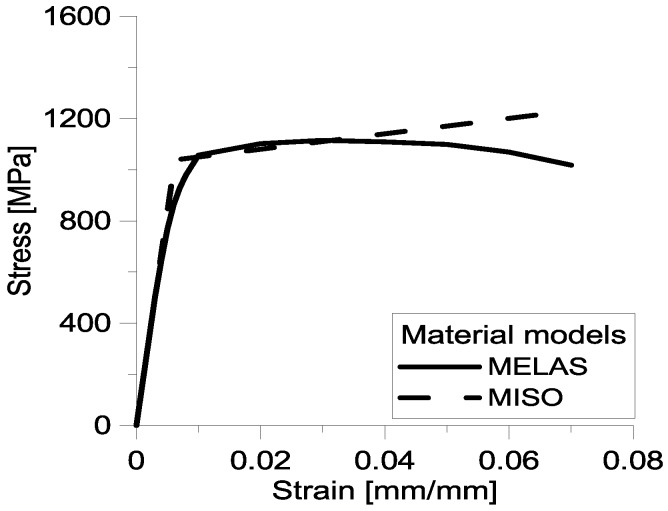
Stress-strain curves corresponding to MELAS and MISO options utilized in the finite element simulations to model the nonlinear constitutive behavior of 18Ni Marage 300.

Compression tests carried out for the six cell variants were simulated by means of the general purpose finite element code ANSYS^®^. FE analysis was basically aimed at assessing the overall constitutive behavior of the micro-cell structures considered in this study. For that purpose, the Multilinear Elastic (MELAS) and Multilinear Isotropic Hardening (MISO) models available in ANSYS were compared. Figure 9 shows the stress-strain data given in input to the finite element model. These data were obtained from experimental compression tests independently carried out on 18Ni Marage 300 specimens following the ASTM standard E9-09 [30]. The MISO curve envelopes the ascending branch of the MELAS curve.

The MELAS model is path independent as it assumes unloading to occur along the same path as loading. Constitutive behavior is described by a piece-wise linear stress-strain curve, with no hysteresis. The MISO model uses the von Mises yield criterion coupled with isotropic work hardening. Constitutive behavior is described by a multilinear stress-strain curve whose initial slope corresponds to the Young’s modulus of the material. This model works well for large strain cycling. The MELAS model is in general much more accurate than the Hookean model but may be less accurate than the MISO model as the latter accounts for plastic deformations and considers the hardening caused by the deformation field imposed to the structure.

**Figure 10:**
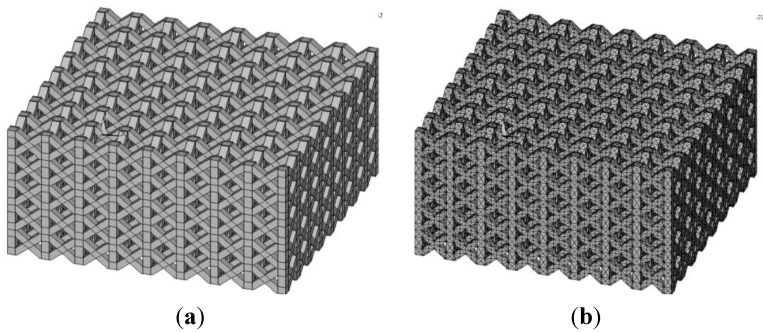
(**a**) 3D model of the pillar texile micro-cellular structure defined in ANSYS; (**b**) Finite element mesh of the 3D model.

FE element models developed for each of the six design variants were meshed with 12-node pyramidal solid elements. The proper element size was selected on the basis of a convergence analysis carried out in order to obtain mesh independent solutions. FE models included on average 197,000 elements and 406,500 nodes. For example, Figure 10 shows the 3D model of the structure geometry defined in ANSYS and the corresponding finite element mesh.

The following kinematic boundary conditions were considered (see Figure 10): the bottom surface of the model cannot displace while the top surface can only move along the vertical direction.

It should be noted that FE analyses were carried out on 3D CAD models that reproduced the theoretical cell structures. Geometric errors made in the SLM process with respect to 3D CAD models were always smaller than 6%. It was assumed that distortions/errors on cell geometry contribute by the same extent to the global error measured on the external surface of the built samples. In order to assess the effect of these geometric errors on the stress/strain distributions predicted by FE analysis, a “distorted” finite element model including geometric errors was developed. Remarkably, differences in structural response with respect to FE models reproducing theoretical structures were always found to be less than 2%.

Porosities and surface roughness also may affect the mechanical response of the cellular structure. However, since in the present case almost full density parts were manufactured via SLM, there was no need to include porosity effects in the numerical analysis. As far as it concerns surface roughness, the average ratio of roughness R_a_ to cell size was about 0.025 (*i.e.*, 15 μm with respect to 500 or 700 μm cell size). Roughness was included as a geometric nonlinearity in the FE model in fashion of surface waviness with an amplitude corresponding to R_a_. Differences in structural response with respect to the theoretical CAD model were still smaller than 5%.

**Figure 11:**
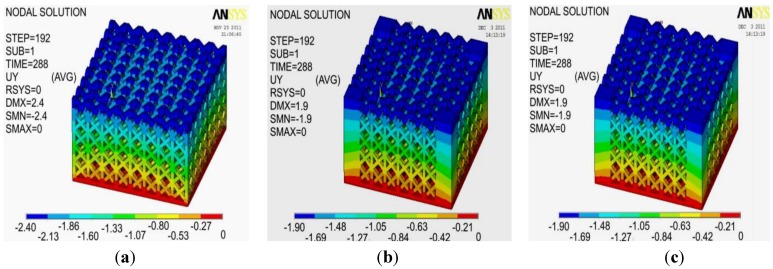
Deformed shapes computed by ANSYS: (**a**) Specimen 1; (**b**) Specimen 2; (**c**) Specimen 3.

Since the mechanical response evaluated by ANSYS was always insensitive to geometric errors and roughness, having used theoretical 3D CAD models in FE computations is indeed reasonable. For example, Figure 11 shows the deformed shapes computed by ANSYS for design A type specimens. In particular, the maps of displacement along the loading direction (*i.e.*, parallel to the height of specimens) are presented. There is a very good agreement between numerical results and the deformed shapes observed experimentally (see Figure 7d–g). Similar results obtained for design B type specimens will be omitted for the sake of brevity.

Table 1 compared the values of peak strength determined from experiments and numerical simulations as the ratio between the peak load and the cross-sectional area of the specimen. Data reported in the table confirm the good agreement between experiments and FE analysis.

**Figure 12:**
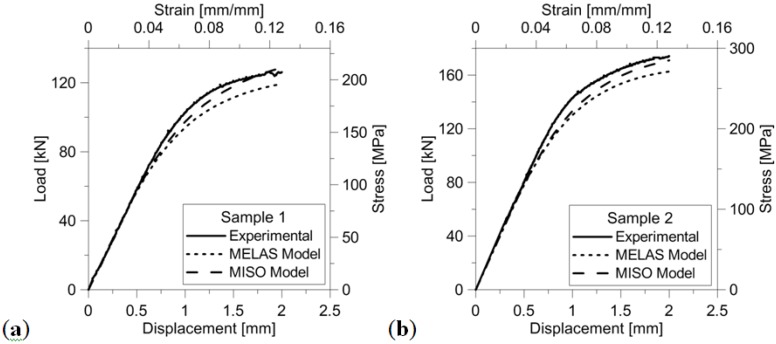

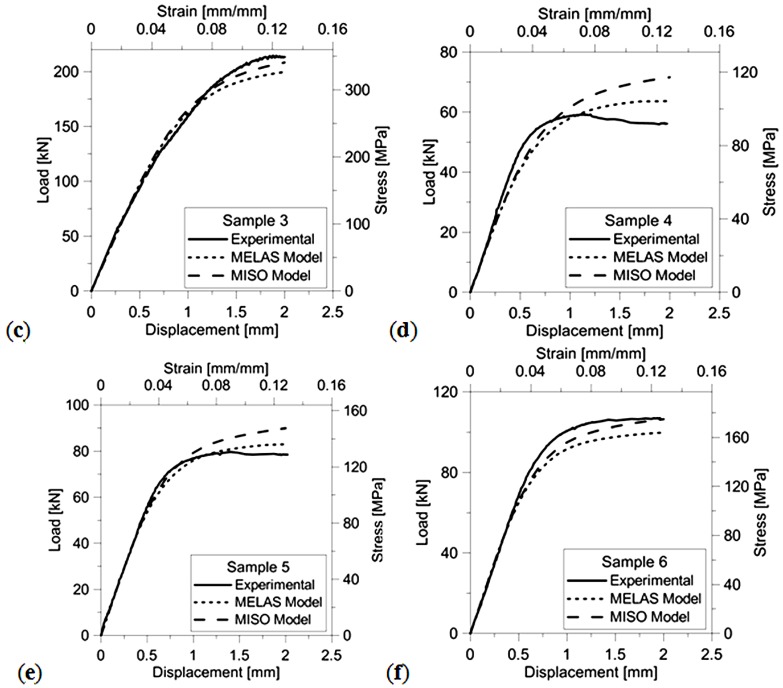
Comparison of the load-displacement and stress-strain curves recorded experimentally and simulated numerically: (**a**) Sample 1; (**b**) Sample 4; (**c**) Sample 2; (**d**) Sample 5; (**e**) Sample 3; (**f**) Sample 6.

Figure 12 compares the load-displacement and stress-strain curves recorded experimentally with those simulated by ANSYS. The MISO model reproduces more accurately the experimentally observed behavior of the design variants. In the linearly elastic range, both models achieve perfect correspondence with experimental results. The difference between the two models becomes less significant as the number of reinforcement columns increases (see also the corresponding values of peak strength reported in Table 1). This is because the presence of columns reduces the risk of plastic buckling. Therefore, numerical results are fully consistent with the experimental evidence of structural failure driven by inelastic buckling phenomena.

However, the finite element models developed in this study might not necessarily capture the unstable behavior caused by surface asperities sharper (*i.e.*, higher values of surface roughness) than in the present case. As the compression load increases, surface asperities can locally collapse and the loading cell can record lower load values (*i.e.*, the steps that are typically observed in the plastic plateau).

## 5. Conclusions

This paper presented a comprehensive study on fabrication and mechanical testing of 18Ni Marage 300 micro-lattice and reinforced micro-lattice components built by means of Selective Laser Melting. The pillar texile micro-lattice structure, comprised of four vertical strut columns and four couples of struts inclined at ±45° with respect to cell axes of symmetry, was selected for being further developed in the second phase of the study as it realizes the best compromise in terms of relative density, area density, and mechanical strength amongst the most commonly used micro-lattice structures described in literature.

SLM was found suitable for manufacturing lattice structures with characteristic dimensions in the micrometer range (the minimum thickness of strut elements that form the cellular structure can be reduced down to 500 μm). Alternative topologies including 4 or 8 reinforcement columns in the micro-lattice structure also were considered.

Compression tests were performed in order to evaluate the mechanical strength under compression of the micro-lattice topology variants built via SLM. Experimental tests were simulated by finite element analyses carried out with the commercial general purpose finite element program ANSYS^®^ Version 13.0 (Canonsburg, PA, USA) in order to assess the constitutive behavior of the lattice structures designed in this study. In particular, two nonlinear stress-strain modeling options available in ANSYS—*i.e.*, Multilinear Elastic (MELAS) and Multilinear Isotropic Hardening (MISO)—were compared to find the most suitable constitutive model.

It was found that the load carrying capability of the structure increases almost linearly with the number of vertical reinforces. For example, by adding 8 reinforcement columns, it is possible to double the load carrying capability of the structure. Remarkably, the increase in load carrying capability is far more significant than the increase in relative density caused by the introduction of vertical reinforcements. This appears to be independent of the characteristic dimensions of the unit cell. The specific strength of the structure hence increases with cell dimensions and strut edge size: this performance indicator is more important than the load carrying capability expressed in terms of peak load before failure.

FE results fully confirmed the inelastic nature of the buckling phenomena driving the failure of the micro-cellular structures analyzed in this study. However, numerical simulations should account for the locally instable behavior caused by the sudden flattening of the sharpest surface asperities that may occur in the case of specimens with highly rough surfaces and introduce steps in the plastic plateau before the nonlinear collapse of the cellular structure. In spite of this limitation, FE analyses carried out in this research reproduced well the nonlinear behavior of very complicated structures such as the micro-cellular designs analyzed in this study.

Overall, the MISO constitutive model reproduced more accurately the experimentally observed behavior of the six design variants considered in this study. As expected, FE results became less sensitive to the modeling option chosen to describe nonlinear constitutive behavior as the number of reinforcement columns increases.
